# A Case Review of Necrotizing Soft Tissue Infection of the Abdomen Utilizing Negative Pressure Wound Therapy with Instillation and Novel Reticulated Open Cell Foam Dressing

**DOI:** 10.7759/cureus.3497

**Published:** 2018-10-26

**Authors:** Stormy Lemay, Elizabeth McElroy, Kersten Reider

**Affiliations:** 1 Wound, Ostomy, Continence Care / Nursing Administration, Reading Health System, Reading, USA

**Keywords:** negative pressure wound, instillation, wound care, necrotizing soft tissue infection, wound cleansing

## Abstract

Necrotizing soft tissue infection is a rapidly spreading bacterial infection that can quickly destroy a person’s muscles, skin, and underlying tissue. In this retrospect chart review, we will look at how the utilization of negative pressure wound therapy with instillation and dwell (NPWTi-d) and novel reticulated open cell foam (ROCF-CC) assisted with the healing of a patient’s wound along with decreasing the time spent in the operating room. NPWTi-d provided the benefits of wound healing such as solubilizing the infectious material and removing the devitalized tissue. Using this form of treatment, we were able to improve the patient’s quality of life and decrease her time in the hospital.

## Introduction

Necrotizing soft tissue infection (NSTI) is the most serious and potentially life-threatening of all skin and soft tissue infections and prompt diagnosis and aggressive treatment are of utmost importance to save patient lives [1]. Standard treatment includes broad-spectrum antibiotics and surgical intervention to remove all the devitalized tissue along with advanced wound care therapy. Effective wound management involves a comprehensive assessment of the patient and of the wound to determine an optimal treatment plan. Wounds at risk for delayed healing include those with extensive tissue loss, critical colonization and/or infection, high levels of exudate, or exposed critical structures [2].

Negative pressure wound therapy (NPWT) is well-known in its success for advanced therapy and treatment in managing wounds, both acute and chronic wounds. NPWT has been reported to increase the rate of healing, promote wound bed granulation, prepare the wound bed for closure, and remove exudate [3]. Wound cleansing is imperative when it comes to wound care. The introduction of fluid into the wound bed is a preventive method used to remove healing impediments, such as cellular debris, necrotic tissue, elevated inflammatory and enzymatic elements, as well as infectious agents [4]. Negative pressure wound therapy with instillation and dwell (NPWTi-d) and negative pressure wound therapy with instillation and dwell with reticulated open cell therapy (ROCF-CC) allows a solution such as normal saline solution (NSS) to be instilled into the wound base to cleanse the wound, dwell within the wound, and then be removed with negative pressure wound therapy. As a result, the planktonic bacterial burden is decreased, contaminants are removed, and the wound is cleansed without manual intervention [5]. This form of therapy has been studied and has shown to increase granulation tissue, decrease trips to the operating room for debridement, reduce a person's length of stay, and decrease the length of treatment.

## Case presentation

A 62-year-old female presented to the emergency department with complaints of abdominal pain and a decrease in urinary output. She has a past medical history of remote colon and endometrial cancers, chronic obstructions, colocutaneous fistulas, diabetes, hypertension, and atrial fibrillation (a-fib). Her surgical history consists of colostomy, right hemicolectomy, hysterectomy, and wound exploration related to the colocutaneous fistula.

**Figure 1:**
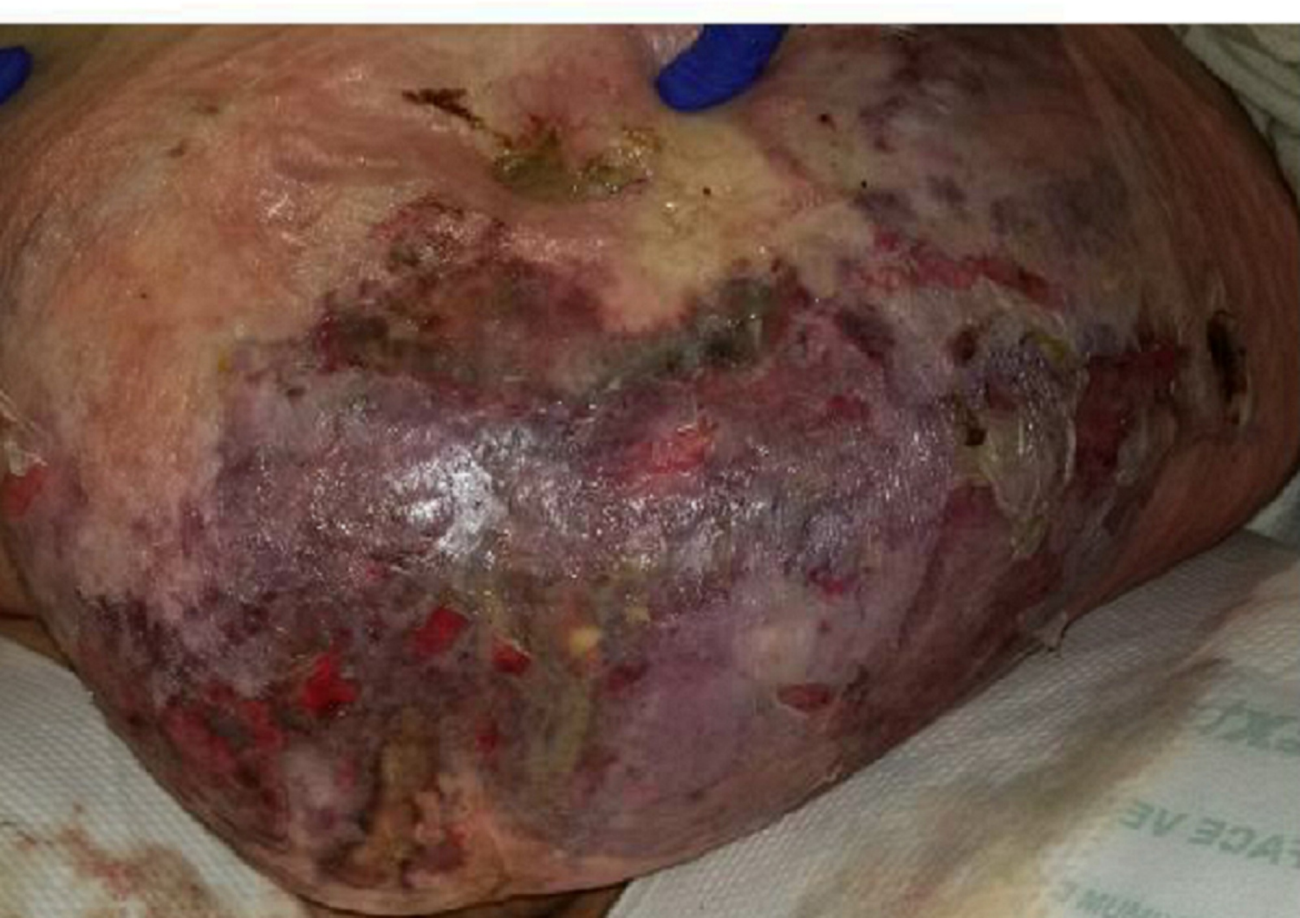
Presentation of abdomen

Upon assessment, she had ventral hernias with a leakage of foul-smelling fluid around her colostomy site. She presented with cellulitis of the entire pannus with some superficial epidermal blistering and necrosis, but the subcutaneous tissue appeared viable, as seen in Figure 1. The computed tomography (CT) scan revealed skin thickening and subcutaneous soft tissue stranding consistent with panniculitis/cellulitis with no drainable abscess. She was started on intravenous vancomycin and cefepime for the treatment of the cellulitis.

Within 24 hours, her abdominal cellulitis had worsened despite receiving broad-spectrum antibiotics. The patient had increased tenderness with palpation to this area. Her white blood cell count (WBC) count increased from 10.2 on admission to 13.4. There was concern for a necrotizing soft tissue infection. She was taken to the operating room that day for emergent debridement. During the debridement, the skin around her hernias was left intact. The surrounding necrotic skin and fat were debrided to the fascia. Her peritoneum was not opened. Tissue samples were obtained, revealing Klebsiella pneumoniae, few Enterococcus faecalis, and Staphylococcus. After debridement, the wound was dressed with betadine-soaked gauze and sterile dressings.

**Figure 2:**
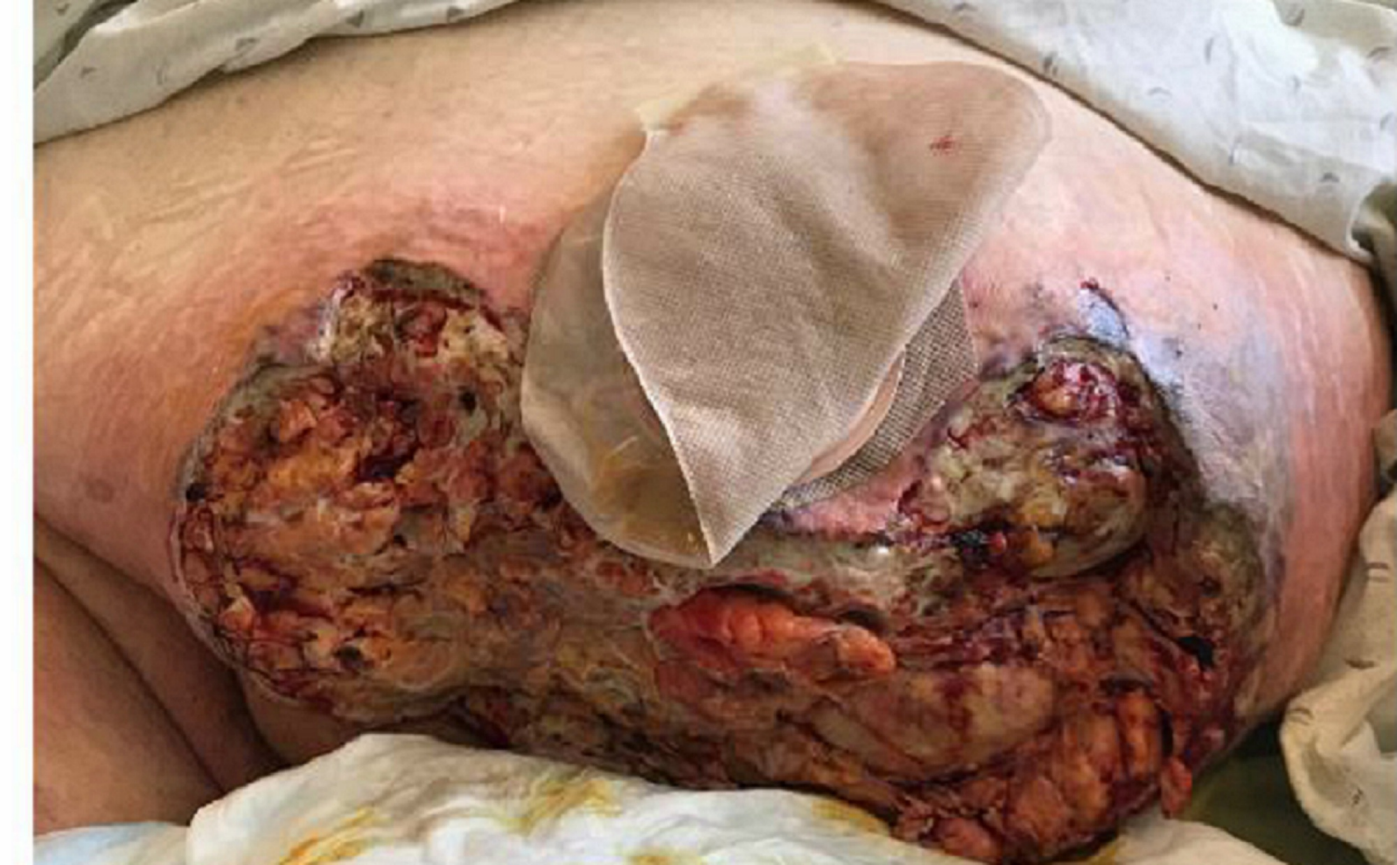
POD 1: Exposed bowel noted to mid-section of abdominal wound POD - post-op day

On post-op day (POD) one, the dressings were removed by the wound, ostomy, continence, nurse (WOCN) team. Upon removal of the dressings, an exposed bowel was noted (Figure 2). There was necrosis noted to the lateral aspects of the wound. The wound was packed with saline-moistened Kerlix and covered in abdominal pads (ABDs). An ostomy appliance was placed over the fistula. The patient was scheduled for further debridement the next day.

On POD two, general surgery performed a wide debridement of the abdominal wall. The enterocutaneous fistula was taken down.

On POD three, she returned to the operating room for another washout and an open abdomen negative pressure therapy (ABThera) was placed. General surgery placed an ABThera with each washout from POD three until POD seven.

On POD seven, a washout was performed and vicryl mesh was placed over the exposed bowel. General surgery applied negative pressure wound therapy (NPWT) using white foam over the mesh followed by black foam.

**Figure 3:**
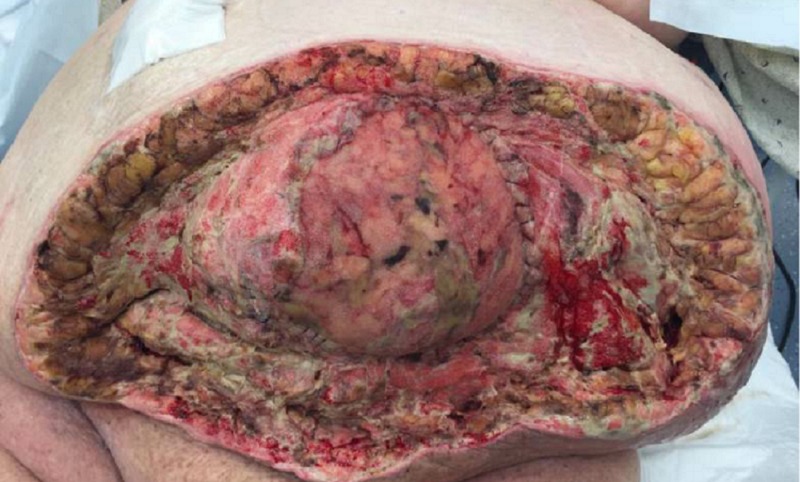
POD 9: Measurements 32 x 54 x 5 cm (8,160 cmᵌ) POD - post-op day

**Figure 4:**
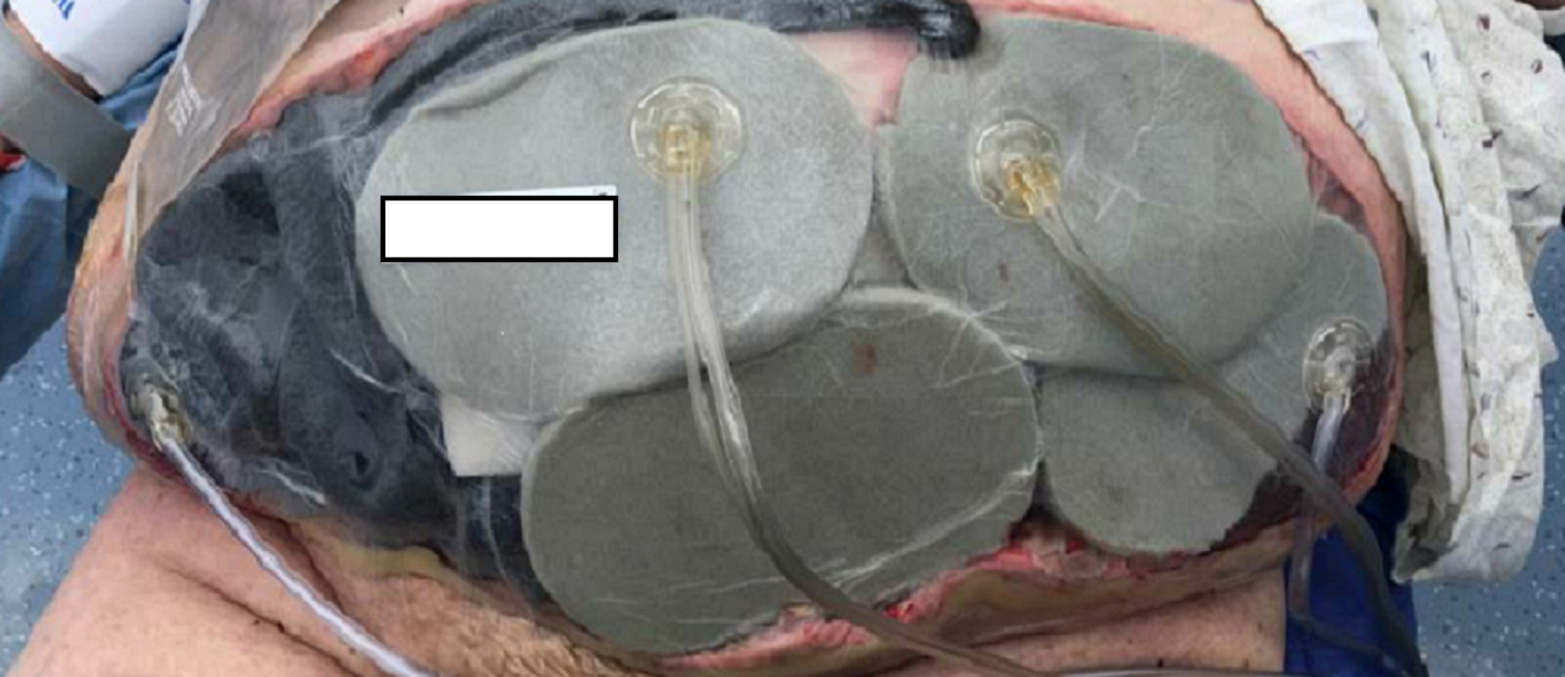
First application of NPWTi-d NPWTi-d - negative pressure wound therapy with instillation and dwell

On POD nine, the patient was taken to the operating room for wound debridement of the skin and subcutaneous fat (Figure 3). After the procedure was completed, an NPWTi-d device was placed in the operating room, as seen in Figure 4. Four barrier rings were placed at the inferior and lateral wound edges to facilitate a good seal and prevent maceration. Four white foam pieces were placed to protect the area with mesh. A total of four kits consisting of both NPWTi-d and ROCF-CC were utilized. The instillation ports were Y connected to the trac pads and applied to four areas in the wound to improve instillation coverage and prevent leakage in the lateral aspects. The NPWTi-d settings were 130 milliliters (ml) of normal saline solution (NSS) to soak for 10 minutes every three and a half hours. Normal saline solution was chosen because it has been shown that despite its direct antimicrobial activity, it can be as effective as other solutions when used with NPWTi-d [6]. The settings for the device were based on the parameters set forth by the review of evidence and recommendations for a cycle frequency of two-four hours at -125 mmHg and a dwell time of 10-20 minutes [7].

On POD 11, the patient returned to the operating room for a washout and dressing change. The NPWTi-d with ROCF-CC was removed. General surgery placed NPWT utilizing white foam over the mesh and covered then with black foam.

**Figure 5:**
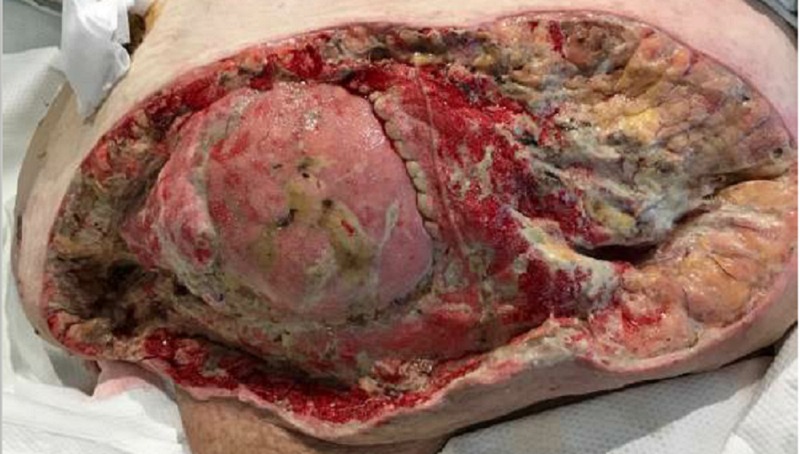
POD 15: 40% pink tissue, 20% red tissue, and 40% slough POD - post-op day

**Figure 6:**
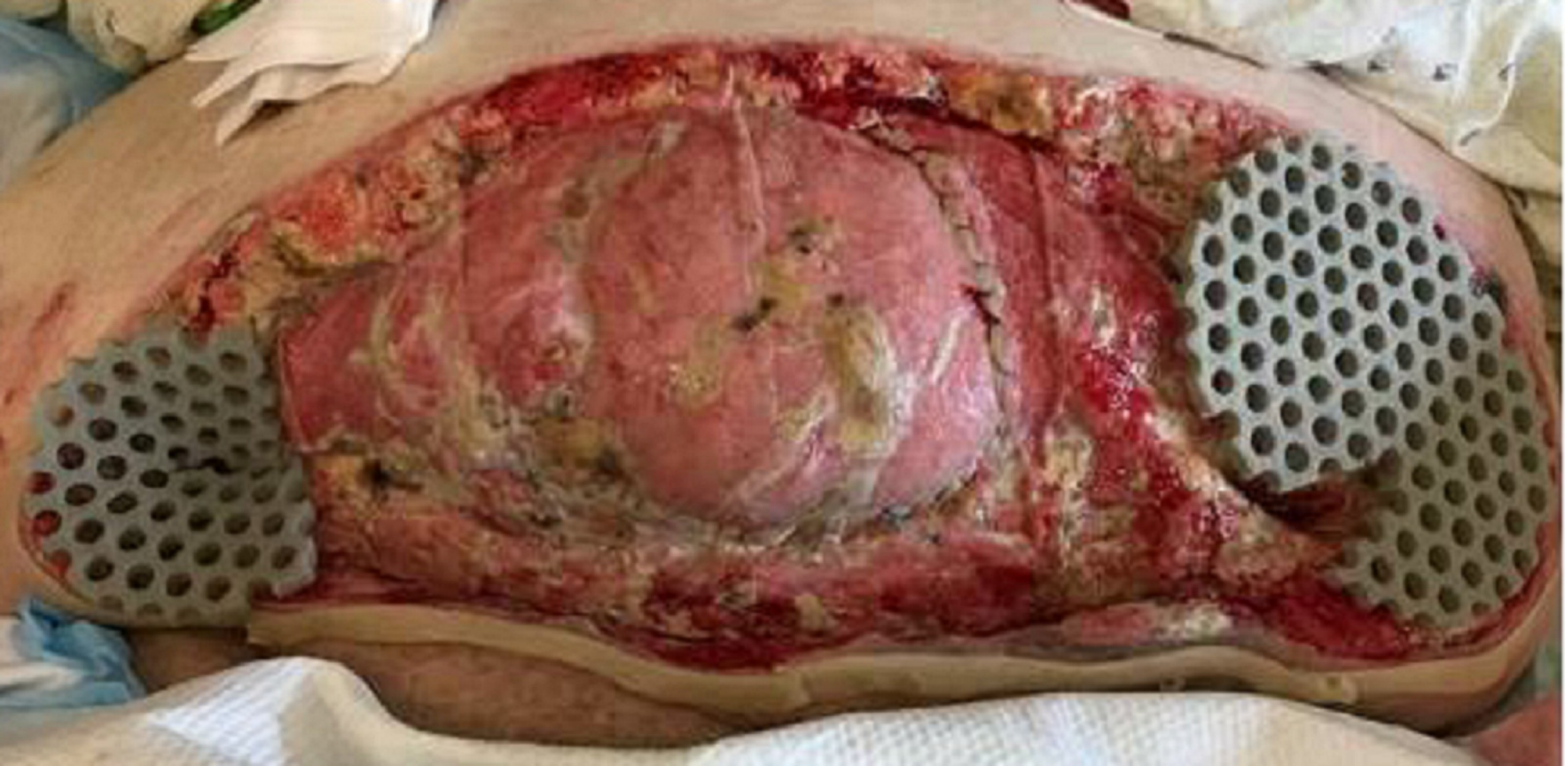
POD 15: Application of ROCF-CC with NPWTi-d. Settings at this time were 130 ml of normal saline set to soak for five minutes every two hours POD - post-op day; ROCF-CC -reticulated open cell therapy; NPWTi-d - negative pressure wound therapy with instillation and dwell

On POD 15, the first NPWTi-d change was performed at the bedside. The wound base had 40% pink tissue, 20% red tissue, and 40% slough, as seen in Figure 5. White foam was placed over the vicryl mesh. NPWTi-d was placed to the central portion. ROCF-CC was applied to the lateral aspects to assist in the removal of the non-viable tissue, as seen in Figure 6. The goal of therapy was to improve granulation tissue and prevent repeated trips to the operating room.

**Figure 7:**
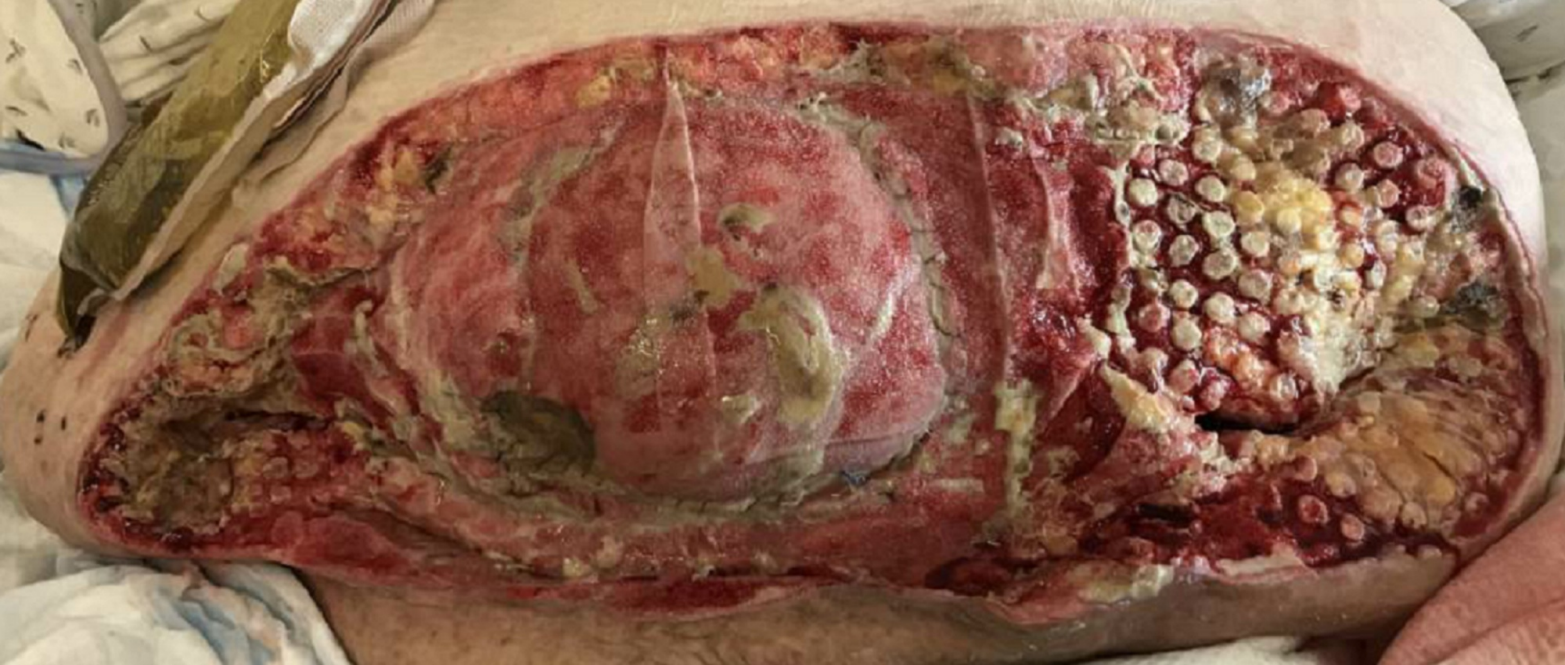
POD 22 abdomen: 40% pink granular tissue, 30% red tissue, and 30% yellow slough POD - post-op day

The patient underwent four dressing changes at the bedside, utilizing NPWTi-d and ROCF-CC. On POD 22, ROCF-CC was discontinued. There was a decrease in devitalized tissue to the lateral aspects. New granulation tissue was present where the macrocolumns are located as seen in Figure 7. White foam was placed over the area with mesh followed by NPWTi-d.

On POD 29, the final dressing change was performed utilizing NPWTi-d. The patient was deemed medically stable for discharge. The receiving facility was unable to accept the patient with NPWTi-d in place. She was transitioned to NPWT, utilizing white foam over the bowel and one NPWT machine.

The patient spent a total of 31 days in the hospital. During this time, she had three ABThera applications, five applications utilizing NPWTi-d, and two applications with NPWT. There was a decrease in devitalized tissue and the wound edges were noted to have contracted. The patient’s measurements reduced from 32 x 54 x 5 cm (8,640 cmᵌ) to 19.5 x 53 x5 cm (5,167 cmᵌ).

**Figure 8:**
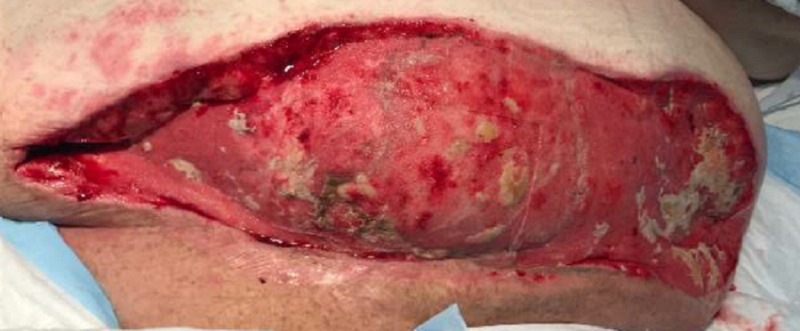
DOT 2 of readmission: 85% red tissue, 15% tan slough, measuring 16 x 54 x 3.5 cm (3,024 cubic cm) DOT - day of treatment

The patient was readmitted to our facility six days after her discharge for altered mentation and possible wound infection. There was concern for purulent drainage noted in the cannister. The dressing was changed the next day in coordination with the surgery and infectious disease teams. Once the dressing was removed, there were no obvious signs of infection. The wound base had 85% red tissue and 15% tan slough, measuring 16 x 54 x 3.5 cm (3,024 cmᵌ) (Figure 8). The patient underwent three dressing changes throughout her readmission. She was discharged to a rehabilitation center where they continued NPWT.

## Discussion

NWPT has been shown to increase the healing time, increase healthy granulation tissue, and can help decrease infection. A study was performed by Morykwas et al. utilizing NPWT in wounds caused by necrotizing fasciitis. Using a pig model, they were able to examine the four responses of wound healing. The study showed that in an animal model, NPWT increased blood flow to and from the wound bed. There was also a significant difference noted in the proliferation of granulation tissue and the tissue bacterial counts had decreased within four days of NPWT [8].

NPWTi-d differs from wound irrigation in that the fluid is instilled slowly and will then remain in the wound for a period of time before being removed by negative pressure. This instillation creates a controlled, protected environment for flushing and cleansing wounds by the proposed mechanism of loosening the contaminants in the wound bed [2]. ROCF-CC is a reticulated open cell foam dressing that includes through holes to facilitate the removal of thick exudate and infectious materials. This dressing can assist with wound cleansing prior to surgical debridement, after surgical debridement, or when surgical debridement is not available or appropriate [9]. NPWTi-d and ROCF-CC were chosen for this patient based on the amount of devitalized tissue there was in the lateral aspects of the wound. This method of treatment permitted quicker wound cleansing. There was a decrease in devitalized tissue from 40% to 15%.

## Conclusions

Necrotizing fasciitis is a rare but severe bacterial infection. If left untreated, or if there is a delay in care, the risk for mortality increases. The gold standard treatment for necrotizing fasciitis includes aggressive wound debridement, antibiotic therapy, and proper wound care. With the use of NPWTi-d and ROCF-CC in combination with surgical debridement and antibiotic therapy, we were able to decrease the patient’s length of stay in the hospital along with decreasing her time in the operating room. Utilizing this treatment plan, a variety of clinicians, including nurses and physicians, were able to collaborate and participate in the care given to the patient.

The authors recognize that this a single case study and that the implications for practice cannot be generalized. This does, however, provide an opportunity for further research to see what settings, what patients, and what clinical diagnosis may benefit the most from the advanced wound therapy of NPWTi-d with ROCF-CC.
